# Prenatal diagnosis and genetic discoveries of an intracranial mixed neuronal-glial tumor: A case report and literature review

**DOI:** 10.1097/MD.0000000000005378

**Published:** 2016-11-11

**Authors:** Lijuan Sun, Qingqing Wu, Yan Pei, Jinghua Li, Jintang Ye, Wenxue Zhi, Yan Liu, Puqing Zhang

**Affiliations:** a Department of Ultrasound, Beijing Obstetrics and Gynecology Hospital, Capital Medical University; b Department of Radiology, Peking University First Hospital; c Department of Pathology; d Department of Obstetrics, Beijing Obstetrics and Gynecology Hospital, Capital Medical University, Beijing, China.

**Keywords:** central nervous system, fetal intracranial tumor, genetics, prenatal imaging, ultrasound

## Abstract

**Background:**

Congenital intracranial tumors as a group are quite rare, representing only 0.5% to 1.5% of all pediatric brain neoplasms.

**Case report:**

We reported a case of congenital mixed neuronal-glial tumor detected by ultrasound at 30 weeks of gestation. It showed that the tumor was 2.5 × 2.3 × 2.1 cm^3^ in size, located in the sellar region, regular shape, and slightly heterogeneous solid mass with a little cystic component. No color flow was present inside the tumor, but the peripheral encirclement by arterial circle of Willis. No other associated malformations were detected. Prenatal magnetic resonance imaging (MRI) which was taken subsequently confirmed the result of ultrasound and provided more detailed information such as fetal brain dysplasia.

The fetal chromosomal karyotype analysis is normal. Single-nucleotide polymorphism (SNP)-based chromosomal microarray analysis (CMA) detected a 0.72-Mb duplication at 4q35.2 in fetus which was associated with epilepsy and cardiac anomalies. It also revealed a 0.13-Mb deletion at 6q26 located in PARK2 gene, and the mutation of the gene is known to be related to autosomal recessive juvenile Parkinson disease.

The parents chose termination of pregnancy (TOP). The histological examination showed a mixed neuronal-glial tumor.

**Conclusion:**

Prenatal detection of mixed neuronal-glial tumor is very rare. Ultrasound is of critical importance to detect the intracranial tumors, and MRI can give us some detailed information about the tumors. However, the precise histologic type was depended on the pathological examination. CMA should be necessary for the fetuses with congenital intracranial tumors, especially when the fetal chromosomal karyotype analysis is normal.

## 1 Introduction

With the development of ultrasound, more and more fetal anomalies can be detected in antenatal period. However, the experience of prenatal diagnosis of fetal intracranial tumors is very limited because of the low incidence. The imaging appearances of various congenital intracranial tumors still overlap, and the final diagnosis still depends on the pathological examination. So far we have found few literatures about prenatal diagnosis of congenital intracranial tumors.

## 2 Case report

Informed consent was obtained from the patient. This case report was approved by the Medical Ethical Committee of Beijing Obstetrics and Gynecology Hospital, Capital Medical University.

A 25-year-old gravida 1 para 0 woman who conceived naturally underwent routine obstetric ultrasonography at 30 weeks’ gestation that revealed an intracranial solid-cystic tumor. The mother suffered from anemia during pregnancy. The father was healthy.

**Figure 1:**
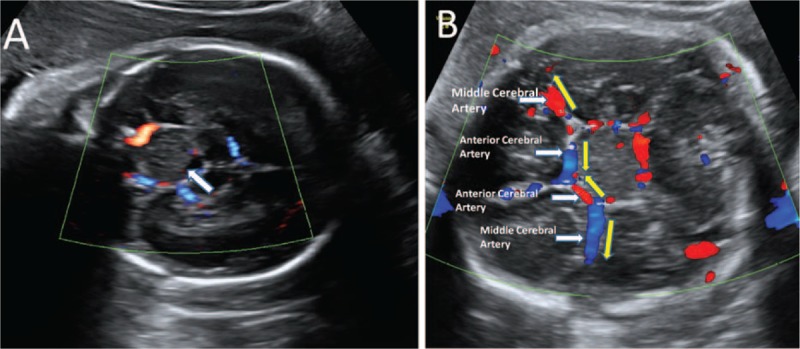
Fetal ultrasonography performed in the 30th week of gestation showed a 2.5 × 2.3 × 2.1 cm^3^ slightly heterogeneous solid mass with a little cystic component (arrowhead) in the sellar region (A) with peripheral encirclement by arterial circle of Willis (B). The red and blue colors indicate the direction of blood flow (yellow arrowhead).

Ultrasound (Fig. 1) showed that the tumor was 2.5 × 2.3 × 2.1 cm^3^ in size, located in the sellar region. It was regular shape and slightly heterogeneous solid mass with a little cystic component. No color flow was present inside the tumor but the peripheral encirclement by arterial circle of Willis. The lateral ventricles and the head circumference were normal. Fetal heart Tei-index was 0.32 (normal). The evaluation of fetal Middle Cerebral Artery (MCA) and umbilical artery (UA) was normal. The amniotic fluid was normal, and no other associated malformations were detected.

**Figure 2:**
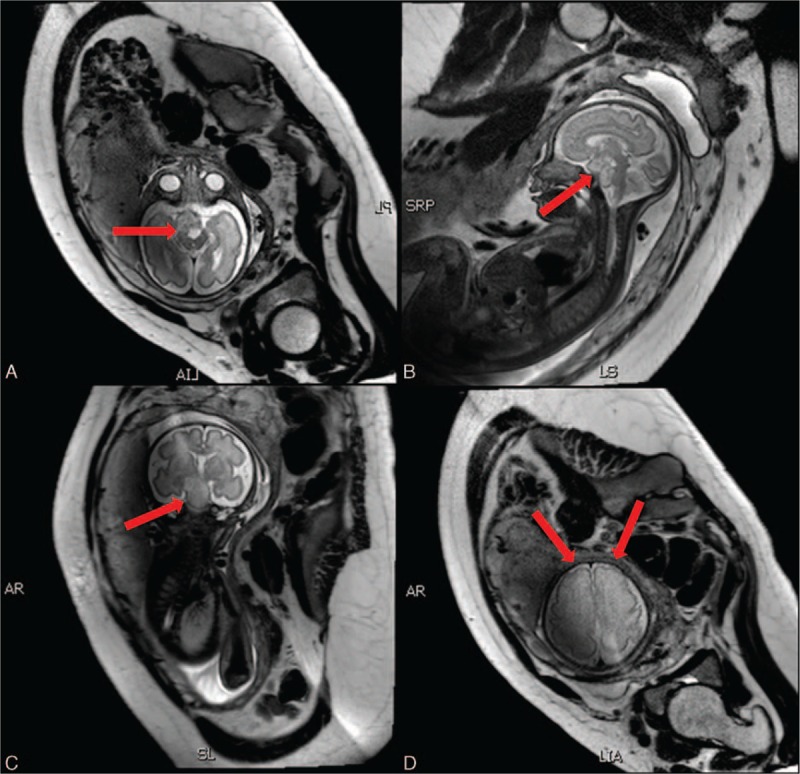
Fetal intracranial tumor was shown (red arrowhead) by axial view (A), sagittal view (B) and coronal view (C) of MRI. Fetal brain dysplasia (D) was diagnosed by the poor developed gyri and sulci of the frontal lobe (red arrowhead). MRI = magnetic resonance imaging.

Magnetic resonance imaging (MRI) which was taken subsequently confirmed the result of ultrasound. Meanwhile, it also provided more detailed information on the fetal central nervous system (CNS) including fetal brain dysplasia and the possible compression of optic nerves caused by the tumor (Fig. 2). The parents were informed that the most likely diagnosis was optical nerve glioma; however, the malignant intracranial tumor was difficult to be excluded prenatally.

**Figure 3:**
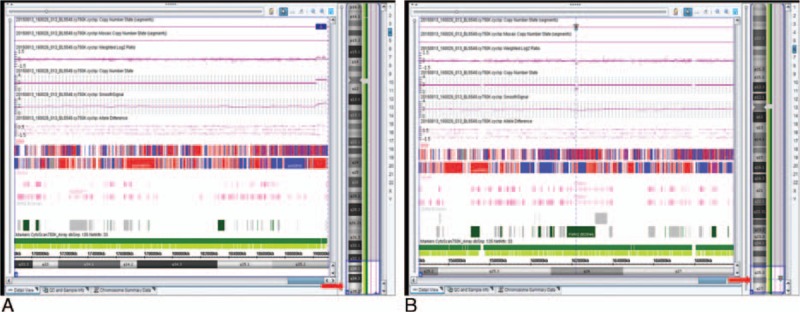
Microarray testing results. (A) A 0.72-Mb duplication at chromosome 4q35.2 (red arrowhead) which encompassed the FRG1 and FRG2 genes. (B) A 0.13-Mb deletion in chromosome 6q26 (red arrowhead), which was located inside the PARK2 gene. The chromosome numbers and cytobands are shown and labeled on the right side. The view on the left side shows the detected segments, regions, and reference annotations in detail. Chromosomal duplication segments are denoted by upward triangle (blue), whereas deletion segments are denoted by downward triangle (red).

The pregnant woman underwent cord blood samplings because of the congenital malformations. Single-nucleotide polymorphism (SNP)-based chromosomal microarray analysis (CMA) (Fig. 3) was performed for prenatal genetic analysis used with fetal cord blood and parental blood samples after the normal chromosomal karyotype analysis was revealed. It detected a 0.72-Mb duplication at 4q35.2 in fetus which was associated with epilepsy (https://decipher.sanger.ac.uk/patient/290426#phenotype/patient-phenotypes) and cardiac anomalies (https://decipher.sanger.ac.uk/patient/288182#phenotype/patient-phenotypes). It encompassed the FRG1 and FRG2 genes. In addition, the CMA also revealed a 0.13-Mb deletion at 6q26 which was located inside the PARK2 gene. The mutation in the PARK2 gene (OMIM ID: ∗602544) is known to be related to autosomal recessive juvenile Parkinson disease.^[1,2]^ Neither the duplication nor deletion was inherited from the parents.

**Figure 4:**
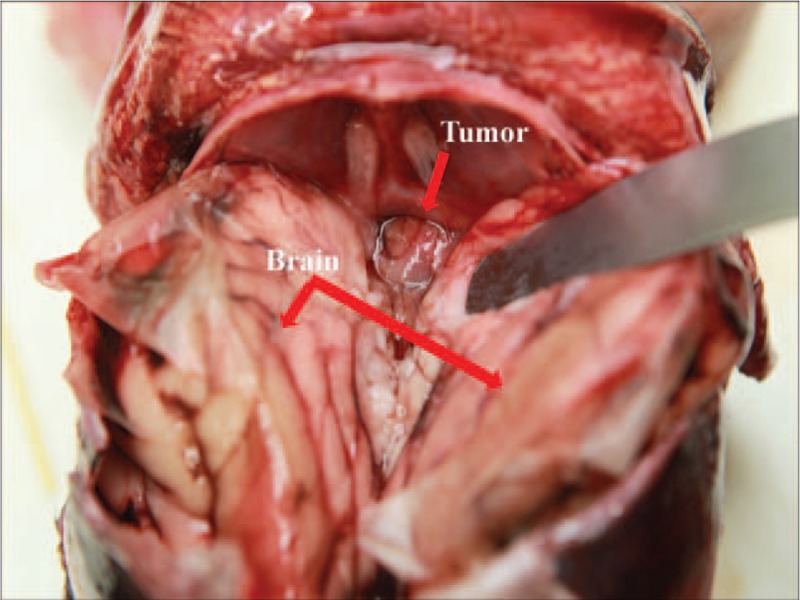
The autopsy showed the brain tumor was in the sellar region (red arrowhead).

Ultimately, the parents chose termination of pregnancy (TOP). The abnormal imaging findings were confirmed by autopsy (Fig. 4). The histological examination showed low-grade neuroepithelial tumor. A mixed neuronal-glial tumor was final diagnosis because 2 cell types (neuronal cells and glial cells) existed in the tumor. The diagnosis was confirmed by immunohistochemistry results.

## 3 Discussion

Congenital intracranial tumors as a group are quite rare, representing only 0.5% to 1.5% of all pediatric brain neoplasms,^[3]^ of which most were congenital intracranial teratomas.^[4–8]^ Prenatal ultrasonography during the whole pregnancy is of particular importance for screening fetal central nervous system tumors. However, few examples of fetal intracranial mixed neuronal-glial tumors have been described by imaging and fewer cases have been confirmed by histopathological examination. Our case contributes to the limited literature focused on the imaging (ultrasonography and MRI), pathological and genetic discoveries of intracranial mixed neuronal-glial tumor in the prenatal period. Searching through the literatures, we only noticed that Chung et al^[9]^ reported a congenital gangliocytoma in 1998 which fell into this category of intracranial mixed neuronal-glial tumor. Its ultrasound features contained both cystic and solid components, located suprasellar and caused marked displacement of the circle of Willis.

We have reviewed the literatures for fetal intracranial tumors which included primarily single case reports published in the last decade assessed by ultrasound (Table 1).^[4,6,10–16]^ Cassart et al^[4]^ retrospectively analyzed imaging findings of congenital craniopharyngioma which was a different pathological type. It showed supra-sellar mass with color flow in its periphery which had the similar sonographic features as our case.

**Table 1:**
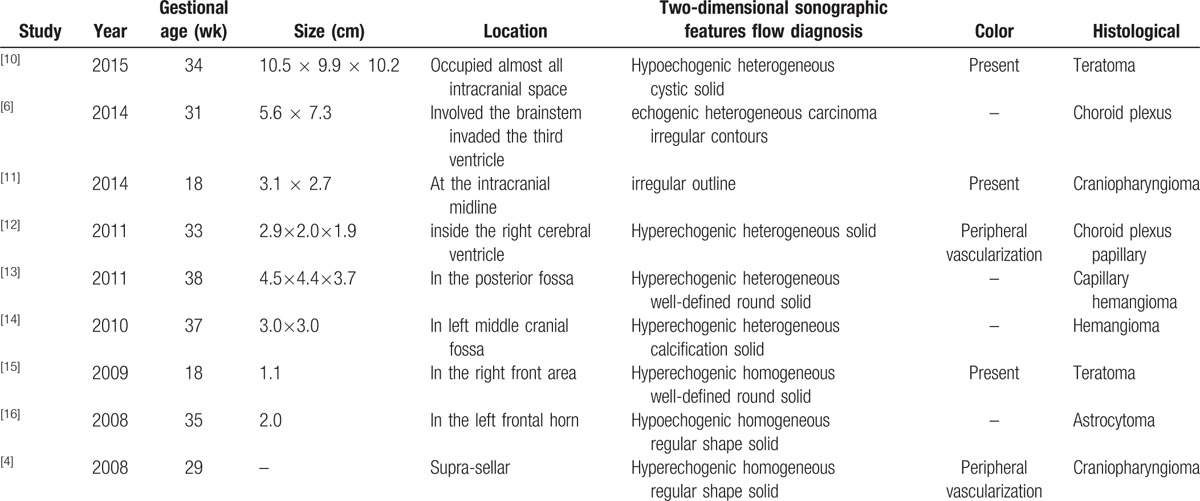
The sonographic features of fetal intracranial tumors of different pathological types.

Notwithstanding the ultrasonography has permitted description of fetal brain anomalies during the antenatal period, the imaging appearances of various congenital intracranial tumors still overlap. Subsequent prenatal MRI allows the confirmation of ultrasound findings and detection for other anomalies that may be present, in particular, intracranial tumor extension.^[8]^ It has been reported that more precise morphology could be provided by MRI at earlier stages of gestation, which makes earlier diagnosis and prompt initiation of treatment possible.^[6]^ Cassart et al^[4]^ noted that MRI was more sensitive than ultrasound for the detection of this heterogeneity. Although there are many advantages of MRI, it must be noted that fetal MRI does not replace ultrasound as a screening tool.

Recently studies on application of CMA for various fetal anomalies have also been published.^[17,18]^ It has been strongly suggested by the early onset of these neoplasms and their embryonal appearance that prenatal factors are important, especially genetic factors.^[19]^ So CMA was performed after fetal chromosomal karyotype analysis was normal. A 0.72-Mb duplication at 4q35.2 was detected in fetus which was associated with epilepsy. It is consistent with the fact that epilepsy is the most common symptom for intracranial neuroepithelial tumor. The CMA also revealed a 0.13-Mb deletion at 6q26 located inside PARK2 gene, and the PARK2 gene mutation has involvement in autosomal recessive juvenile Parkinson disease.^[1,2]^ The clinical phenotypes of this disease included shaking palsy, slow moving and myodystony, and so on. However, the microduplication at 4q35.2 and the microdeletion at 6q26 were still defined as being of uncertain clinical significance.

## 4 Conclusion

Prenatal detection of mixed neuronal-glial tumors is very rare. Ultrasound and MRI are helpful for diagnosing intracranial tumors, but the precise histologic type of the tumor was depended on pathological examination. CMA should be necessary for the fetuses with congenital intracranial tumors. This finding not only provides information for clinical consultation but may also allow more accurate genetic diagnosis.
